# MEIS2 suppresses breast cancer development by downregulating IL10

**DOI:** 10.1002/cnr2.2064

**Published:** 2024-05-06

**Authors:** Yongzhi Xiao, Yingzhe Liu, Yangqing Sun, Changhao Huang, Shangwei Zhong

**Affiliations:** ^1^ Department of Ultrasound Diagnosis, The Second Xiangya Hospital Central South University Changsha Hunan China; ^2^ Xiangya International Medical Center, National Clinical Research Center for Geriatric Disorders, Xiangya Hospital Central South University Changsha Hunan China; ^3^ Department of Oncology, Xiangya Hospital Central South University Hunan China; ^4^ The Cancer Research Institute, Hengyang Medical School University of South China Hengyang China

**Keywords:** breast cancer, IL10, MEIS2, myeloid cells

## Abstract

**Background:**

Breast cancer (BC) is the most commonly diagnosed female cancer. Homeobox protein MEIS2, a key transcription factor, is involved in the regulation of many developmental and cellular processes. However, the role of MEIS2 in the development of breast cancer is still unclear.

**Aims:**

We aimed to examine the role of myeloid ecotropic insertion site (MEIS2) in breast cancer and the association of MEIS2 with breast cancer clinical stages and pathological grades. We revealed the underlying mechanism by which MEIS2 affected breast cancer cell growth and tumor development.

**Methods and Results:**

Using human BC cell lines, clinical samples and animal xenograft model, we reveal that MEIS2 functions as a tumor suppressor in breast cancer. The expression of MEIS2 is inversely correlated with BC clinical stages and pathological grades. MEIS2 knockdown (MEIS2‐KD) promotes while MEIS2 overexpression suppresses breast cancer cell proliferation and tumor development in vitro and in animal xenograft models, respectively. To determine the biological function of MEIS2, we screen the expression of a group of MEIS2 potential targeting genes in stable‐established cell lines. Results show that the knockdown of MEIS2 in breast cancer cells up‐regulates the IL10 expression, but MEIS2 overexpression opposed the effect on IL10 expression. Furthermore, the suppressive role of MEIS2 in breast cancer cell proliferation is associated with the IL10 expression and myeloid cells infiltration.

**Conclusion:**

Our study demonstrates that the tumor suppressor of MEIS2 in breast cancer progression is partially via down regulating the expression of IL10 and promoting myeloid cells infiltration. Targeting MEIS2 would be a potentially therapeutic avenue for BC.

## 1 INTRODUCTION

Homeodomain‐containing family of proteins show fundamental functions in the regulation of numerous developmental and cellular processes.^1^, ^2^ Based on the three‐dimensional structure of the homeodomain, homeodomain proteins have been classified into six distinct groups.^1^ Among them, the TALE (three‐amino‐acid loop extension) is an atypical subfamily as the proteins of which have three extra amino acids residues proline‐tyrosine‐proline in its first loop region.^1^, ^3^ TALE subfamily includes pre‐B cell leukemia homeobox (PBX) proteins and myeloid ecotropic insertion site (MEIS) proteins, which form complexes with HOX proteins and function as cofactors of HOX proteins.^1^, ^3^, ^4^, ^5^, ^6^

The homeobox protein MEIS2 belongs to the MEIS family. MEIS2 acts as a key transcription factor that regulates cell differentiation and embryonic development. MEIS2 knockout mice are embryonic lethal.^7^, ^8^, ^9^, ^10^, ^11^ The expression of MEIS2 is deregulated in some cancers, and MEIS2 functions differently in cancers of different tissue (organ) origins.^12^, ^13^, ^14^, ^15^ In prostate cancer, MEIS2 shows a negative role in the regulation of a constitutive intrinsic inflammatory signaling circuit that promotes castration‐resistant prostate cancer (CRPC) development. Downregulation of MEIS2 in prostate cancer cells activates the signaling circuit and promotes CRPC development.^12^, ^16^, ^17^, ^18^ However, in acute myeloid leukemia (AML)‐ETO‐positive leukemia, in which MEIS2 is up‐regulated, MEIS2 plays an oncogenic role through impairing repressive DNA binding of AML1‐ETO, resulting in upregulation of proto‐oncogenes such as YES1 kinase in AML cells.^19^ The discrepancy of MEIS2 expression and role in different cancers suggests that MEIS2 is not a universal oncogene or tumor suppressor, and the role of MEIS2 in cancer depends on the signaling networks that MEIS2 is involved in. Gene expression analysis has shown that MEIS2 expression is associated with the proliferation of breast cancer (BC) cells,^20^ but the role of MEIS2 in BC and its underlying mechanism remain elusive.

Tumor microenvironment (TME), composed of numerous stromal cells, factors and extracellular matrix, plays crucial roles in tumor development, progression, metastasis and therapeutic resistance.^21^ Myeloid derived suppressor cells (MDSCs) is a major cellular component of TME, contributing to the immunosuppressive environment in tumor site.^22^ MDSCs can secret various growth factors to promote (breast) cancer cell growth and affect the interactions between immune effectors and cancer cells; meanwhile, (breast) cancer cells can secret cytokines and chemokines to recruit myeloid cells into TME, and then ‘educate’ them into potent immunosuppressive cells to support tumor progression.^23^, ^24^, ^25^, ^26^, ^27^

Here, we show that the MEIS2 expression in BC cells is associated with BC clinical stages and pathological grades. Knockdown of MEIS2 expression promotes BC cells growth, while overexpression of MEIS2 suppresses BC development. The underlying mechanism may be that MEIS2 affected breast cancer cell proliferation, migration, as well as the infiltration of myeloid cells in BC by regulating the IL10 expression. The tumor suppressor role of MEIS2 is partially mediated by downregulation of IL10.

## 2 MATERIALS AND METHODS

### 2.1 Cells and reagents

Breast cancer cell lines (T47D and MDA‐MB‐231) were purchased from American Type Culture Collection (ATCC). Cells were cultured as previously described.^28^ Cells being transfected with the lentiviral stocks to stably express MEIS2 shRNA (Forward sequence: CCGGGAGCCAAGGAGCAG CATATAGCTCGAGCTATATGCTGCTCCTTGGCTCTTTTTG, Reverse sequence: AATTCAAAAAGAGCCAAGGAGCAGCATATAGCTCGAGCTATAT GCTGCTCCTTGGCTC), IL10 shRNA (Forward sequence: CCGGGCC TACATGACAATGAAGATACTCGAGTATCTTCATTGTCATGTAGGCTTTTTG, Reverse sequence: AATTCAAAAAGCCTACATGACAATGAAGATACTCGAG TATCTTCATTGTCATGTAGGC) or control shRNA (pLKO.1 empty vector) were cultured in DMEM supplemented with 10% FBS and 10 μg/mL puromycin. Cells stably expressing control vector (pcDNA), pcDNA‐MEIS2c, were cultured in DMEM containing 10% FBS and G418 (1000 μg/mL). Flag‐tag antibody and HA‐tag were purchased from Cell Signaling Technology.

### 2.2 Cell viability assay

Cell viability assay was conducted as previously described.^16^ In brief, 96‐well plates were used to seed cells. The MTT dye solution was added into each well, after culturing to different time‐points. Then plates were incubated at 37°C for 4 h. The solubilization Solution/Stop Mix was added into each well before measured, and 96‐well plate reader was used to record the absorbance of each plate at wavelength of 570 nm with 630 nm as reference wavelength. And the absorbance at 570 nm minus the absorbance at 630 nm is the value of cell viability.

### 2.3 Scratch wound healing assay

The cells were seeded in six‐well plates. When the cells were a confluent monolayer, we used a sterile 200 pipette tip to wound the cell monolyers, and then washed twice by PBS to remove cell debris before imaging with microscope. The cell migration rate = (wound distance at 0 h − wound distance at indicated time)/wound distance at 0 h.

### 2.4 Real time polymerase chain reaction analysis

The mRNA expression levels of MEIS2 and its associated‐genes were quantified using real‐time polymerase chain reaction (qRT‐PCR) analyses. Briefly, Qiagen RNeasy Mini Kit (Qiagen) was used to purify total RNA from cells according to the manufacturer's protocol. The reverse transcription was conducted with high‐capacity cDNA Reverse Transcription Kit (Thermo Fisher) to obtain cDNA from the extracted RNA. SYBR Green qPCR Supermix (Solis BioDyne) was used for the real‐time PCR according to the manufacturer's protocol on BIO‐RAD iQ5 real‐time PCR Detection System (BIO‐RAD). The mRNA levels of MEIS2 and its potentially regulating genes were normalized to the housekeeper gene GAPDH. Graphpad Prism 5 software was applied to perform Two‐tailed and unpaired *t*‐tests. The sequences of primers used for real‐time PCR were as following: MEIS2‐F: GAAAAGGTCCACGAACTGTGC, MEIS2‐R: CTTTCATCAATGACGAGGTCGAT; IL10‐F: GACTTTAAGGGTTA CCTGGGTTG, IL10‐R: TCACATGCGCCTTGATGTCTG;GAPDH‐F: AAGGT GAAGGTCGGAGTCAAC, GAPDH‐R: GGGGTCATTGATGGCAACA ATA.

### 2.5 Western blot

SDS‐PAGE gel was applied to separate whole cell lysates, and then the proteins were transferred to polyvinylidene fluoride membranes (PVDF) (Millipore), subsequently being blocked in 5% nonfat milk solution for 1 h. Immunostaining was conducted using specific primary antibodies for MEIS2 (Abcam), HA‐tag (Cell Signaling Technology), β‐Actin (Santa Cruz), and Flag‐tag (Cell Signaling Technology) at 4°C overnight. After HRP‐conjugated second antibody incubation, the Pierce ECL Western Blotting Chemoluminescence Reagents (Thermo Scientific) was applied for the detection.

### 2.6 Immunofluorescence

The paraffin‐embedded human breast tumor tissue sections were stained with anti‐CD33 (Cell Signaling Technology) and anti‐CD11b (Invitrogen). Before staining, antigen retrieval solution (Sigma) was used to treat the tissue sections (5 μm thick). Then the samples were incubated with the specific primary antibodies in 10% blocking serum overnight at 4°C after being blocked in 10% serum solution. After washing thrice with PBS at the next day, AF594 secondary antibodies and Fluorescein (FITC) (Jackson ImmunoResearch Laboratories) were applied for 30 min. Then the samples were incubated with DAPI for 5 min at RT, and a fluorescence microscope (Leica) were applied to capture images.

### 2.7 Paraffin‐embedded human BC samples

The study was approved by the Medical Ethics Committee of the Second Xiangya Hospital, Central South University, and the informed consent was obtained from all the patients involved with the tissue samples. Paraffin‐embedded specimens were from patients with BC who had surgical treatment from September 2012–2015 at the Second Xiangya Hospital. The pathological and clinical data of these patients were obtained from the records retrospectively of patients.

### 2.8 Immunohistochemistry

The paraffin‐embedded human breast tumor tissue sections were stained with anti‐MEIS2 (Abcam). The immunohistochemistry (IHC) was performed as the procedure previously described.^16^ In brief, before being stained by using a Vectastain® abc kit (Vector Laboratories), antigen retrieval solution (Sigma) was used to treat the paraffin‐embedded tissue sections. After being blocked by 10% blocking serum, the tissue sections were incubated with the anti‐MEIS2 at 4°C overnight, then next incubated with biotinylated secondary antibody and avidin‐biotinylated peroxidase complex (ABC kit) (VECTOR LABORATORIES), respectively. Finally, 3,3’‐diaminobenzidine (DAB) (VECTOR LABORATORIES) was used to stain the tissue sections, and then the sections was counterstained with hematoxylin. The MEIS2 protein primarily located in the nuclear of tumor cells. The Nuclear staining intensity (NI) was noted as 0 (negative), 1 (weak), 2 (moderate), and 3 (strong); and the nuclear staining fraction (NF) was assigned a score of 0 (0%–5%), 1 (6%–25%), 2 (26%–50%), 3 (51%–75%), or 4 (>75%). A combined Nuclear score (NS) was calculated by multiplying NI and NF (range of 0–12).

### 2.9 Transwell migration assay

For measurement of myeloid cell migration, GFR Matrigel (BD Biosciences) was applied to coat transwell inserts (Greiner Bio‐One ThinCert™ CellCoat™, Fisher Scientific). Briefly, the BC cells transfected with sh‐MEIS2 or/and sh‐IL10, sh‐control lentrivirus were seeded in the lower well. DMEM media supplemented with vehicle or IL10 neutralization antibody (anti‐IL10) was added to the lower well. U937 cells (1.5 × 10^3^) were grown in DMEM media in the upper side of the insert. After 48 h, the invading cells were fixed, and then stained, imaged and analyzed.

### 2.10 Animal studies

The 6 week‐old NOD‐SCID female mice were used for BC xenograft model. To investigate the role of MEIS2 in BC development, female mice were subcutaneously inoculated with 4 × 10^6^ MDA‐MB‐231 cells expressing MEIS2 (MEIS2‐OE) or control vector (Control) as well as 1 × 10^6^ MDA‐MB‐231 cells expressing MEIS2 shRNA (MEIS2‐KD) or shRNA (Control). Cells were mixed with GFR Matrigel (Corning), and then inoculated into NOD‐SCID female mice subcutaneously. Tumor growth was measured and monitored as indicated.

### 2.11 Quantification and statistical analysis

The Student's *t* test was used to examine the statistical significance of differences between indicated groups. analysis of variance (ANOVA) is applied when more than two groups are compared for the statistically significant analysis. All *p* values were two‐tailed, when *p* < .05, it was considered statistically significant (*p < .05; **p < .01; ***, *p* < .001).

## 3 RESULTS

### 3.1 The expression of MEIS2 is reversely correlated with BC pathological grades and BC differentiation

**FIGURE 1:**
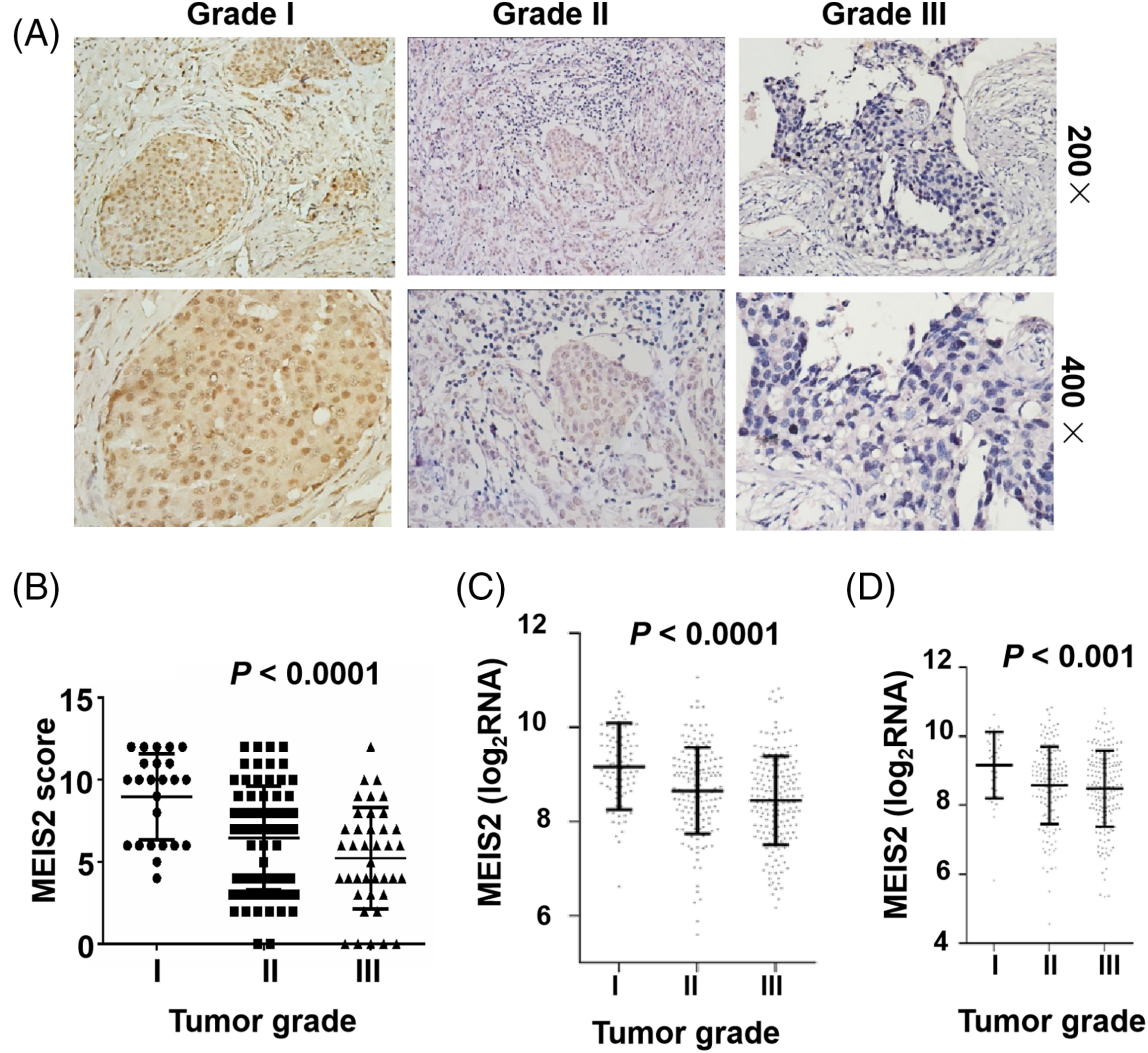
Myeloid ecotropic insertion site (MEIS2) expression in human breast cancer is associated with tumor grades and BC differentiation. (A) Immunohistochemistry (IHC) analysis for MEIS2 expression in paraffin‐embedded tissue sections of human breast cancer at different tumor grades. (B) The comparison of MEIS2 expression examined by IHC in different grade of human breast cancers. The data are presented as mean ± SEM, and the significance was calculated by the ANOVA. (C,D) The comparison of MEIS2 mRNA expression in different grade of human breast cancers. Gene array data is obtained from GSE78958 (*N* = 424) (C) and GSE86166 (*N* = 366) (D).

To define the role of MEIS2 in BC, the expression level of MEIS2 in paraffin‐embedded sections of 107 cases of human BC samples was assayed by immunohistochemistry (IHC). Our results showed that the staining of MEIS2 was mostly in nuclei of BC cells, the strong MEIS2 staining was observed in the grade I tumors, whereas the staining became weaker in grade II and further weaker in grade III tumors, which showed less differentiation in BC compared with grade I tumors (Figure 1A). The expression of MEIS2 in BC is significantly and reversely correlated with tumor pathological grades and BC differentiation (Figure 1B). In addition, we analyzed the MEIS2 expression in BC in two different datasets of Gene Expression Omnibus (GEO). The GSE78958 microarray dataset contained gene expression profiles from 424 cases of BC specimens with different tumor grades,^29^ and the GSE86166 had the gene expression profiles with clinicopathological data of 366 cases of BC patients.^30^ Consistently, comparative analysis of these patient‐derived datasets showed a reverse correlation between MEIS2 expression and BC tumor pathological grades (Figure 1C,D).

### 3.2 The expression of MEIS2 in BC is reversely related to clinical tumor node metastasis (TNM) stages of BC

**FIGURE 2:**
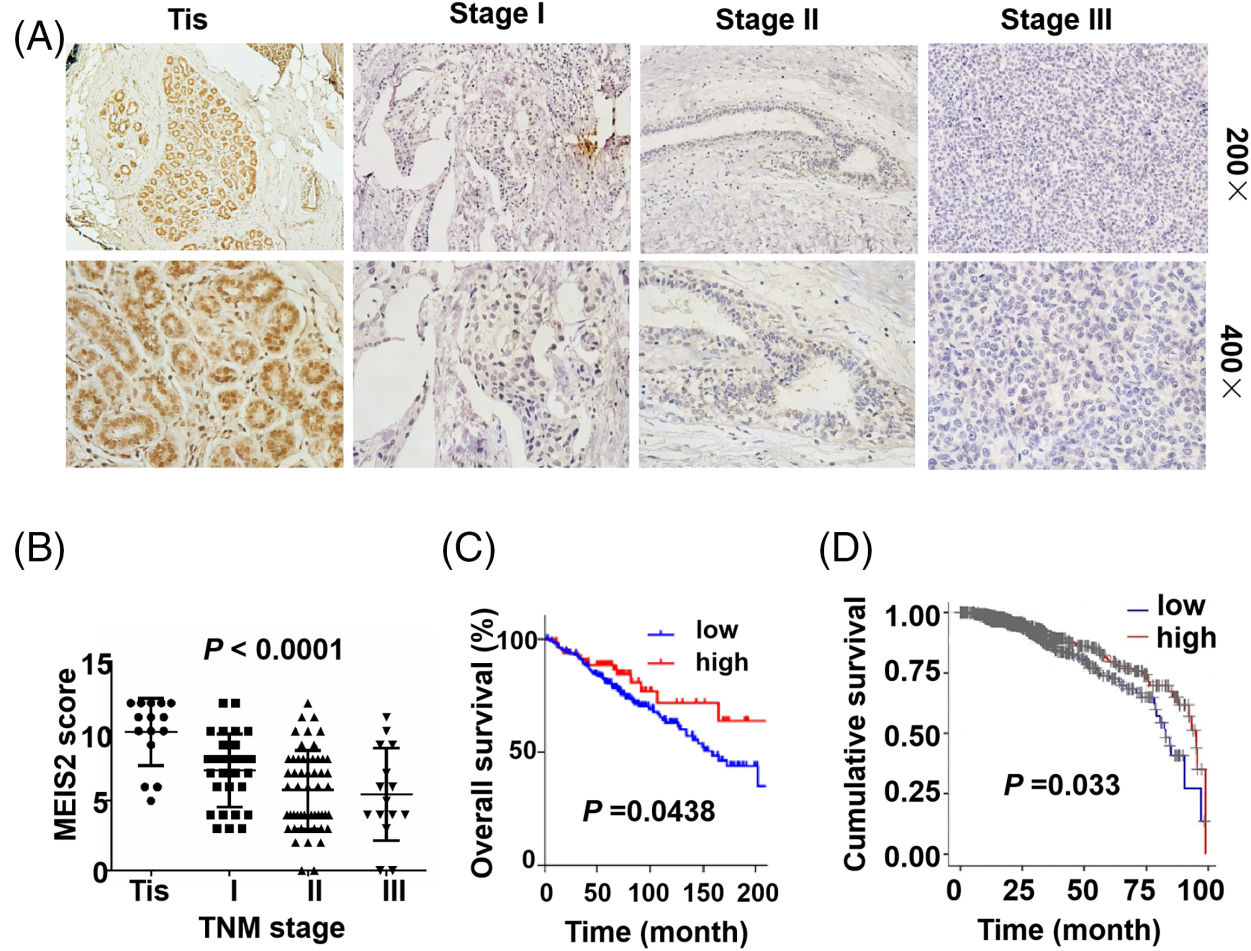
Myeloid ecotropic insertion site (MEIS2) expression in human breast cancer is reversely correlated with clinical tumor node metastasis (TNM stages). (A) IHC analysis for MEIS2 expression in paraffin‐embedded tissue sections of human breast cancer at different TNM stages. (B) The comparison of MEIS2 expression examined by IHC in different clinical TNM stages of human breast cancers. The data are presented as mean ± SEM, and the significance was calculated by the analysis of variance. (C) Kaplan–Meier overall survival curves for breast cancer patients with different levels of MEIS2 expression. Gene array data is obtained from GSE78958 (*N* = 424). (D) Kaplan–Meier survival plots for the patients with different MEIS2 expression levels obtained from TIMER.

We also analyzed the expression of MEIS2 in different stages of human BC samples, and found that MEIS2 was strongly expressed in BC cells of ductal carcinoma in situ (DCIS) (TNMs tage Tis), while the expression level of MEIS2 was decreased in stage I tumors and further decreased in stage II and III tumors (Figure 2A). The expression level of MEIS2 was significantly and reversely correlated with BC clinical TNM stages (Figure 2B). In addition, we sought to identify whether the MEIS2 expression in BC is associated with overall survival (OS) rates of BC patients. We analyzed the Kaplan–Meier (KM) overall survival curves for the MEIS2 expression in two datasets of BC,^31^ and found that the OS time of patients with high expression of MEIS2 was significantly longer than that with low expression (Figure 2C,D). These results indicate that MEIS2 expression in BC is closely associated with BC progression, and may be a potential biomarker for prognosis of BC.

### 3.3 Knockdown of MEIS2 promotes BC development and growth

**FIGURE 3:**
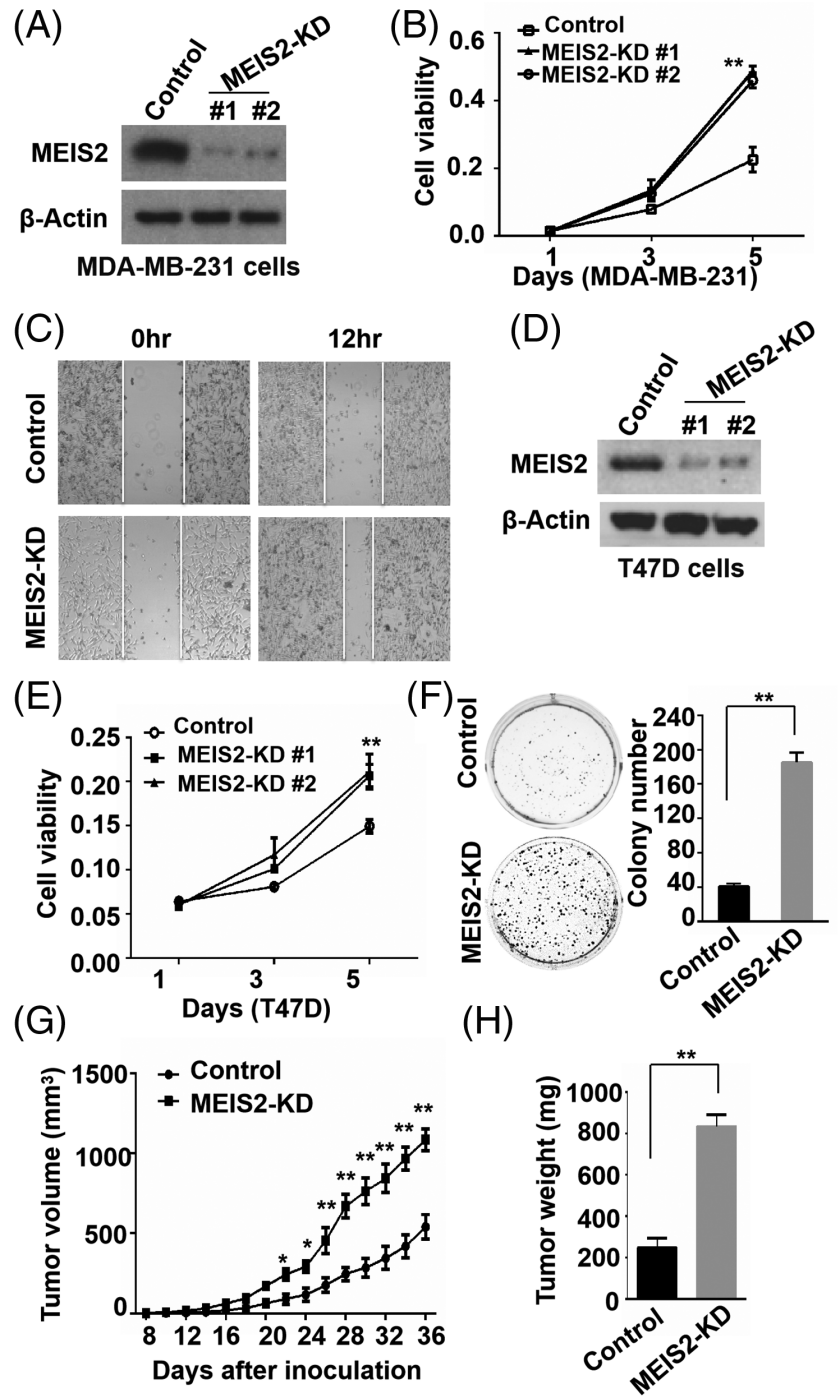
Knockdown of myeloid ecotropic insertion site (MEIS2) promotes breast cancer growth and development. (A) Western blot for MEIS2 expression in control and MEIS2 knockdown (MEIS2‐KD) MDA‐MB‐231 cells. (B) MTT assay for cell proliferation rates of control and MEIS2‐KD MDA‐MB‐231 cells. (C) Representative images of scratch wound‐healing assay for control and MEIS2‐KD MDA‐MB‐231 at 0 and 12 h after wound scratch. (D) Western blot for MEIS2 expression in control and MEIS2‐KD T47D cells. (E) MTT assay for cell proliferation rates of control and MEIS2‐KD T47D cells. (F) Representative images of colony formation for control and MEIS2‐KD T47D cells. (G) Tumor development curve of xenograft mouse model inoculated with control and MEIS2‐KD MDA‐MB‐231 cells (*n* = 5). (H) Weight comparison of tumors (*n* = 5) collected from mice in (G) 36 days after cell inoculation.

To further define the role of MEIS2 in BC development, we established control and MEIS2 stable knockdown (MEIS2‐KD) BC cells by transfecting MDA‐MB‐231 and T47D cells with lentiviruses engineered expressing MEIS2 or control shRNA, and selected by puromycin treatment. The expression of MEIS2 in these clones were confirmed by Western blot (WB) (Figure 3A,D). MTT assay was applied to measure the cell proliferation rates. We found the amount of MEIS2 knock‐down breast cancer cells is much more than the control group after 5 days, indicating that knockdown of MEIS2 significantly increased the proliferation rates of both MDA‐MB‐231 and T47D MEIS2‐KD cells as compared with their control cells (Figure 3B,E). And MEIS2 silence enhanced the ability of mobility and colony formation in breast cancer cells (Figure 3C,F). To examine the role of MEIS2 in tumor development in animal models, 1 × 10^6^ control or MEIS2‐KD MDA‐MB‐231 cells were mixed with Matrigel (1:1), and then inoculated *s.c*. into NOD‐SCID female mice, tumor development was monitored. We found that MEIS2 knockdown significantly promoted MDA‐MB‐231 xenograft tumor growth in mouse models (Figure 3G,H). These results indicate that MEIS2 functions as a tumor suppressor in BC.

### 3.4 Overexpression of MEIS2 suppresses BC growth and tumor development

**FIGURE 4:**
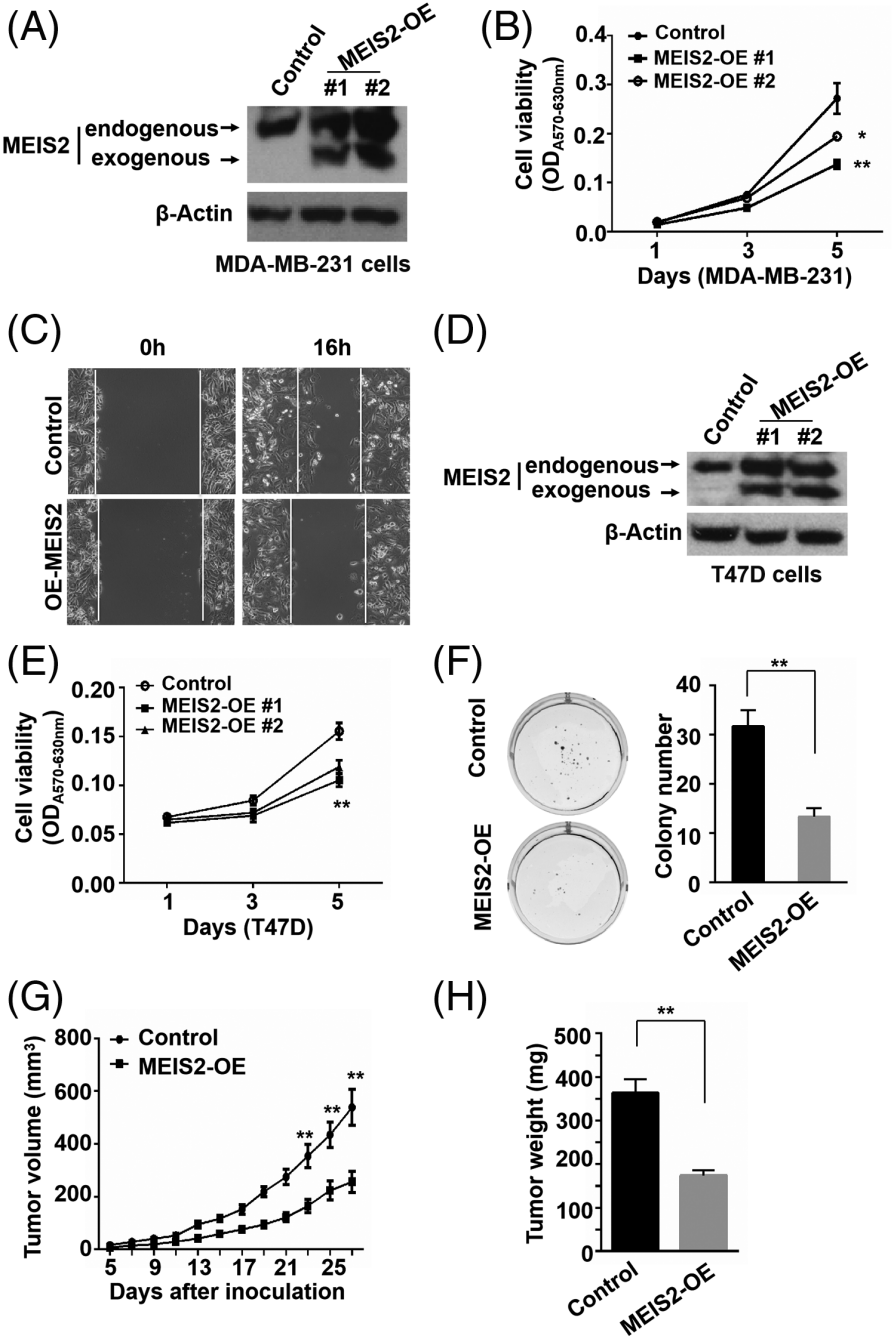
Overexpression of myeloid ecotropic insertion site (MEIS2) suppresses breast cancer growth and development. (A) Western blot for MEIS2 expression in control and MEIS2 overexpression (MEIS2‐OE) MDA‐MB‐231 cells. (B) MTT assay for cell proliferation rates of control and MEIS2‐OE MDA‐MB‐231 cells. (C) Representative images of scratch wound‐healing assay for control and MEIS2‐OE MDA‐MB‐231 cells at 0 and 16 h after wound scratch. (D) Western blot for MEIS2 expression in control and MEIS2‐OE T47D cell lines. (E) MTT assay for cell proliferation rates of control and MEIS2‐OE T47D cells. (F) Representative images of colony formation for control and MEIS2‐OE T47D cells. The results represent the mean ± SEM of three independent experiments performed in triplicate. (G) Tumor development curve of xenograft mouse model inoculated with control and MEIS2‐OE MDA‐MB‐231 cells (*n* = 5). (H) Weight comparison of tumors (n = 5) collected from mice in (G) 26 days after cell inoculation.

To further confirm the role of MEIS2 in BC, MEIS2 stable overexpression (MEIS2‐OE) and control BC cells were established by transfecting MDA‐MB‐231 and T47D cells with control or MEIS2 (transcript variant I) expression plasmid, and selected by G418 treatment. The expression of MEIS2 in these clones were confirmed by Western blot (Figure 4A,D). MTT assay was applied to analyze the cell proliferation rates. We found that overexpression of MEIS2 significantly decreased the proliferation rates of both MDA‐MB‐231 and T47D MEIS2‐OE cells compared with their control cells (Figure 4B,E). And MEIS2 overexpression suppressed the ability of mobility and colony formation of BC cells compared with their control cells (Figure 4C,F). To detect the role of MEIS2 in tumorigenicity of BC cells in animal models, 4 × 10^6^ control or MEIS2‐OE MDA‐MB‐231 cells mixed with Matrigel (1:1) were inoculated *s.c*. into NOD‐SCID female mice, tumor development was monitored. We found that overexpression of MEIS2 significantly suppressed BC xenograft tumor development in mouse models (Figure 4G,H). These results further demonstrated the suppressive role of MEIS2 in BC development.

### 3.5 MEIS2 mediates breast cancer cell proliferation and the infiltration of MDSCs through IL10

**FIGURE 5:**
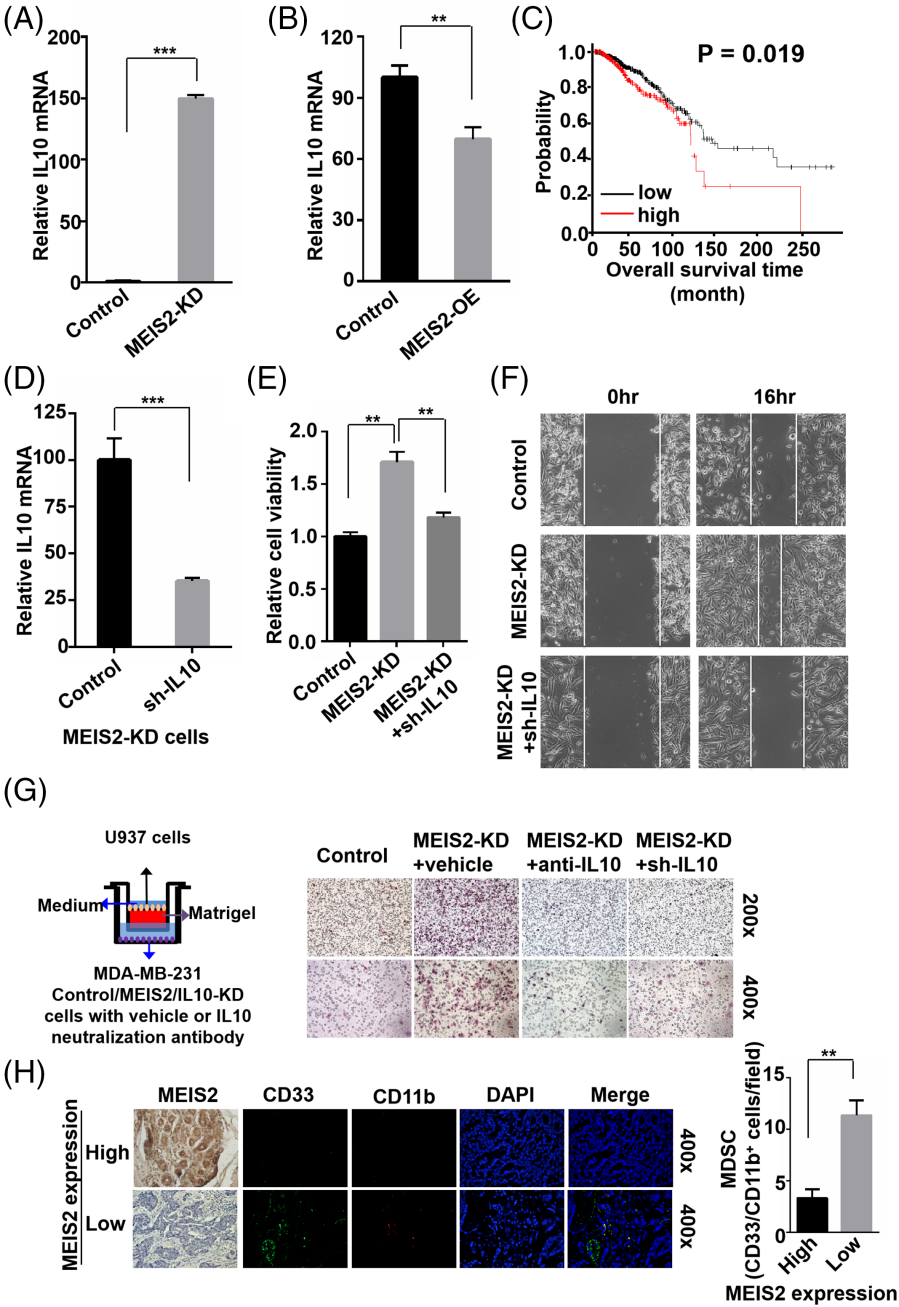
Myeloid ecotropic insertion site (MEIS2) mediates breast cancer cell proliferation and the infiltration of myeloid‐derived suppressor cells (MDSCs) through IL10. (A,B) Real‐time PCR analysis for the mRNA expression of IL10 in control and MEIS2‐KD (A)/MEIS2‐OE (B) MDA‐MB‐231 cells. The data are presented as mean ± SEM, and the significance was calculated by the Student's *t* test. (C) Kaplan–Meier survival plots for the breast cancer patients with different IL10 expression levels obtained from the Kaplan Meier plotter. (D) Real‐time PCR analysis for the efficiency of IL10 knockdown in MEIS2‐KD MDA‐MB‐231 cells. (E) MTT assay for the effect of MEIS2 or/and IL10 knockdown on the cell proliferation rates of MDA‐MB‐231 cells. (F) Representative images of scratch wound‐healing assay for the effect of MEIS2 or/and IL10 knockdown on MDA‐MB‐231 cells at 0 and 16 h after wound scratch. (G) Representative images of transwell migration assay for U937 cells cultured in the upper chamber, and control, MEIS2‐KD knockdown MDA‐MB‐231 cells cultured in lower chamber. For the MEIS2‐KD groups: (1) MEIS2‐KD MDA‐MB‐231 cells transfected with IL10 knockdown lentivirus, (2) vehicle or (3) 1 μg/mL IL10 neutralization antibody (anti‐IL10) was added in lower chamber as indicated in the figure. (H) Representative images of immunofluorescence analysis for the expressions of CD33 and CD11b protein, the marker for MDSCs cells, in high/low MEIS2 expression breast cancer tissue sections.

To investigate the mechanisms by which MEIS2 affects BC growth and development, we searched MEIS2 target genes based on MEIS2 protein DNA binding motif in Harmonizome database and TFBS, a transcription factor target gene database.^32^, ^33^ The regulation of these potential genes by MEIS2 was further screened by real‐time PCR analysis in control and MEIS2‐KD MDA‐MB‐231 cells. Our results showed that a group of genes was differentially expressed in control and MEIS2‐KD MDA‐MB‐231 cells, and IL10 was one of the most differentially expressed genes (Figure S1). The real‐time PCR analysis showed that the expression level of IL10 is highly upregulated in MEIS2 knockdown cancer cells, while significantly decreased in MEIS2 overexpressed cells, indicating that MEIS2 can affect the IL10 expression level in breast cancer cell (Figure 5A,B). According to the Kaplan Meier plotter database, IL10 expression level was correlated with poor clinical prognosis of BC (Figure 5C). Therefore, we hypothesized that MEIS2 affected cancer cell growth through regulating the expression of IL10. To test this hypothesis, we silenced the IL10 in MEIS2‐KD cells (Figure 5D), and then we applied MTT assay to investigate the effect of IL10 silence on BC cell proliferation. The results showed that IL10 silence alleviated the effects of MEIS2 on mobility and proliferation rate of BC cells (Figure 5E,F), indicating that MEIS2 mediated the proliferation rate and mobility of cancer cells through IL10.

In addition, MEIS2‐IL10 signaling pathway was associated with the infiltration of myeloid cells. The result of transwell assay demonstrated that MEIS2 silence enhanced the migration of myeloid cells, while IL10 silence or IL10 neutralization antibody (anti‐IL10) treatment can reduce the MEIS2 associated infiltration of myeloid cells (Figure 5G). Consistently, the amount of MDSCs in MEIS2 low expression human breast cancer sample was much more than in MEIS2 high expression samples (Figure 5H). Together, these results indicate that the functions of MEIS2 is partially mediated through regulation of IL10 expression in BC.

## 4 DISCUSSION

In women, breast cancer (BC) is a commonly diagnosed cancer and a leading cause of cancer death worldwide, and there is about 2.3 million new diagnosed BC cases (about 11.7% of the total new cancer cases) and 0.68 million BC deaths worldwide in 2020.^34^ Although there are some advances in the mechanism of BC devolopment and some therapeutic strategies being developed for BC, the treatment of BC remains a clinical challenge.^35^, ^36^, ^37^ To develop novel therapeutic approaches for this disease, it is needed to identify new genes and signaling pathways that are crucial to BC development and progression. In present study, our studies demonstrate that the expression of MEIS2 in BC is associated with BC clinical stages and pathological grades, and MEIS2 functions as a tumor suppressor in BC. Furthermore, our studies suggest that MEIS2 downregulates the expression of IL10 to suppress breast cancer growth, and MEIS2‐IL10 signaling is partially associated with MDSCs infiltration in BC. MEIS2 may be a potential prognostic biomarker and a therapeutic target for BC.

MEIS2 is an important member of the atypical subfamily of homeobox genes. As a key transcription factor, MEIS2 is implicated in regulating numerous developmental and cellular processes, such as cell proliferation and differentiation, through affecting various signaling networks.^8^, ^38^, ^39^ Some evidences suggest that MEIS2 is associated with some cancers progression. However, the exact role of MEIS2 in different cancers is not unified and seems to be tissue‐type dependent.^12^, ^13^, ^14^, ^15^ For examples, MEIS2 functions as an oncogene in AML and ovarian cancer.^13^, ^19^ However, in prostate cancer, MEIS2 shows a negative role in the regulation of the constitutive intrinsic inflammatory signaling circuit that promotes the development castration‐resistant prostate cancer (CRPC).^12^, ^16^, ^17^ Maybe it seems contradictory, the possible reason is that MEIS2 involves in more than one signal network, by which MEIS2 conducts its specific role in a certain cancer. In AML, the reason behind MEIS2 acts as an oncogene is that MEIS2 can directly bind to the Runt domain of AML1‐ETO to impair repressive DNA binding of AML1‐ETO, resulting in the upregulation of proto‐oncogene YES1.^19^ While, in prostate cancer, MEIS2 is an important component of a constitutive intrinsic inflammatory signaling circuit, that controls the constitutive NF‐κB activation in CRPC cells.^16^ Here, we demonstrate that MEIS2 acts as a suppressor in breast cancer (ER− and ER+), which is partially due to the MEIS2‐IL10 signaling. Therefore, it would be a good chose to suppress BC development by interrupting this signaling.

The cytokines/chemokines derived from cancer cells or/and stromal cells are closely associated with the formation of immunosuppressive environment in tumor site. Interleukin‐10 (IL‐10) is a regulatory cytokine with low molecular weight, and is associated with (breast) cancer initiation and progression, but the effect of IL10 on (breast) cancer development is complex.^40^ IL10 can be produced by both immune cells and (breast) cancer cells.^41^, ^42^ IL10 is known as an anti‐inflammatory cytokine and can induce immunosuppression to help cancer cells to escape from immune surveillance.^40^, ^43^ Here, we found that MEIS2 can regulate the expression level of IL10 to affect the infiltration of myeloid cells, which may be helpful to understand why IL10 facilitates the formation of immunosuppressive niche in tumor. Our results indicate that MEIS2 might involve in the remodel of TME in tumor site, but whether MEIS2 affects other immune cells such as T cell in TME is also very interesting. Additionally, MEIS2‐mediated IL10 contributes to breast cancer cell growth, knockdown IL10 significantly reduced the proliferation rate of MEIS2‐KD breast cancer cells. The potential mechanism may be that IL10 silencing causes apoptosis in BC cells.^42^

In sum, our study revealed that the homeobox protein MEIS2 acts as a tumor suppressor in the development of breast cancer (ER− and ER+). MEIS2 mediates breast cancer cell growth and the infiltration of myeloid cells by regulating IL10 expression. The expression level of MEIS2 is associated with breast cancer clinical stages and pathological grades, suggesting its potential value for breast cancer prognostics. Furthermore, targeting the dysfunction of MEIS2 might be a promising strategy for BC therapy.

## AUTHOR CONTRIBUTIONS

**Yongzhi Xiao:** Conceptualization; methodology; writing – original draft. **Yingzhe Liu:** Conceptualization; methodology; writing – original draft. **Yangqing Sun:** Methodology; writing – original draft; conceptualization. **Changhao Huang:** Conceptualization; methodology; writing – original draft. **Shangwei Zhong:** Conceptualization; methodology; investigation; writing – original draft; writing – review and editing; funding acquisition.

## CONFLICT OF INTEREST STATEMENT

The authors have stated explicitly that there are no conflicts of interest in connection with this article.

## ETHICS STATEMENT

The study was approved by the Medical Ethics Committee of the Second Xiangya Hospital, Central South University, and the informed consent was obtained from all the patients involved with the tissue samples.

## Supporting information


**Appendix S1.** Supporting Information.

## Data Availability

The data that support the findings of this study are available from the corresponding author upon reasonable request.
