# Supplementary material for: MEIS2 suppresses breast cancer development by downregulating IL10
Source: Cancer Rep (Hoboken). 2024 May 6;7(5):e2064. doi: 10.1002/cnr2.2064 (PMC11074520; doi:10.1002/cnr2.2064)
Supplement: Supplementary file 1 — Appendix S1. Supporting Information. [file CNR2-7-e2064-s001.doc]

**Supporting information**

**MEIS2 Suppresses Breast Cancer Development by Downregulating IL10**

Yongzhi Xiao^1^, Yingzhe Liu^2^, Yangqing Sun^3^, Changhao Huang^3^, Shangwei Zhong^4*^


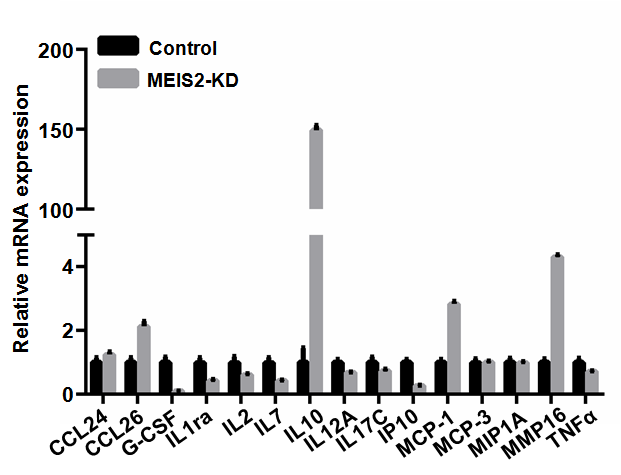


**Figure S1** Analysis of the expression of potential genes in control and MEIS-KD MDA-MB-231 cells.

**Table S1** Correlation of MEIS2 expression with clinicopathologic characteristics of breast carcinomas.

| **Characteristic** | **MEIS2 expression** | |
| --- | --- | --- |
|  | **Low expression No. (%)** | **High expression No. (%)** |
| Age |  |  |
| T-stage |  |  |
| Tis | 0(0) | 11(10.28%) |
| T1 | 10(9.34%) | 25 (23.36%) |
| T2 | 26(24.30%) | 28(26.17%) |
| T3 | 3(2.8%) | 4(3.74%) |
|  |  |  |
| N-stage |  |  |
| N0 | 19（17.76%） | 34（31.78%） |
| N1 | 19（17.76） | 26（24.3） |
| N2 | 4（3.74%） | 4 （3.74%） |
| N3 | 1 （0.93%） | 0（0%） |
| Histologic grade |  |  |
| 1 | 7（6.54%） | 25（23.36%） |
| 2 | 22（20.56%） | 34（31.78%） |
| 3 | 11（10.28%） | 8（7.48%） |

Note: Among the 107 BC samples, high MEIS2 protein expression (combined NS >4) was detected in 66 samples (61.68%), and low MEIS2 protein expression (combined NS ≤4) was observed in 41 samples (38.32%).
